# The Genome of a *Bacillus* Isolate Causing Anthrax in Chimpanzees Combines Chromosomal Properties of *B. cereus* with *B. anthracis* Virulence Plasmids

**DOI:** 10.1371/journal.pone.0010986

**Published:** 2010-07-09

**Authors:** Silke R. Klee, Elzbieta B. Brzuszkiewicz, Herbert Nattermann, Holger Brüggemann, Susann Dupke, Antje Wollherr, Tatjana Franz, Georg Pauli, Bernd Appel, Wolfgang Liebl, Emmanuel Couacy-Hymann, Christophe Boesch, Frauke-Dorothee Meyer, Fabian H. Leendertz, Heinz Ellerbrok, Gerhard Gottschalk, Roland Grunow, Heiko Liesegang

**Affiliations:** 1 Centre for Biological Security (ZBS), Robert Koch-Institut, Berlin, Germany; 2 Research Group Emerging Zoonoses (NG2), Robert Koch-Institut, Berlin, Germany; 3 Goettingen Genomics Laboratory, Institute of Microbiology and Genetics, Georg August University Goettingen, Goettingen, Germany; 4 LANADA/Laboratoire Central de Pathologie Animale, Bingerville, Côte d'Ivoire; 5 Department of Primatology, Max Planck Institute for Evolutionary Anthropology, Leipzig, Germany; University of Hyderabad, India

## Abstract

Anthrax is a fatal disease caused by strains of *Bacillus anthracis*. Members of this monophyletic species are non motile and are all characterized by the presence of four prophages and a nonsense mutation in the *plcR* regulator gene. Here we report the complete genome sequence of a *Bacillus* strain isolated from a chimpanzee that had died with clinical symptoms of anthrax. Unlike classic *B. anthracis*, this strain was motile and lacked the four prohages and the nonsense mutation. Four replicons were identified, a chromosome and three plasmids. Comparative genome analysis revealed that the chromosome resembles those of non-*B. anthracis* members of the *Bacillus cereus* group, whereas two plasmids were identical to the anthrax virulence plasmids pXO1 and pXO2. The function of the newly discovered third plasmid with a length of 14 kbp is unknown. A detailed comparison of genomic loci encoding key features confirmed a higher similarity to *B. thuringiensis* serovar konkukian strain 97-27 and *B. cereus* E33L than to *B. anthracis* strains. For the first time we describe the sequence of an anthrax causing bacterium possessing both anthrax plasmids that apparently does not belong to the monophyletic group of all so far known *B. anthracis* strains and that differs in important diagnostic features. The data suggest that this bacterium has evolved from a *B. cereus* strain independently from the classic *B. anthracis* strains and established a *B. anthracis* lifestyle. Therefore we suggest to designate this isolate as “*B. cereus* variety (var.) anthracis”.

## Introduction

The Bacillus cereus group comprises six species, *Bacillus cereus*, *Bacillus thuringiensis*, *Bacillus anthracis*, *Bacillus weihenstephanensis*, *Bacillus mycoides* and *Bacillus pseudomycoides*. These species are closely related, and the strains of *B. cereus* sensu stricto, *Bacillus thuringiensis*, and *Bacillus anthracis* share highly conserved chromosomes but differ in the virulence encoding plasmids [1]. Whereas *B. thuringiensis* is an insect pathogen [2], *B. cereus* is known mainly as a food poisoning bacterium able to cause diarrhea and vomiting, but is also able to cause more severe infections [3]. *B. anthracis*, the etiological agent of anthrax, is found worldwide and is able to infect virtually all mammals. It is a matter of debate whether these bacteria represent three distinct species or are subspecies of *B. cereus* sensu lato [4], [5]. The species-specific phenotype and pathogenicity are often plasmid-encoded [1], [6], like the toxins and capsule of *B. anthracis* [7], the insecticidal crystal proteins of *B. thuringiensis* [8], and the cereulide synthesis of emetic *B. cereus* strains [9]. However, other virulence factors like hemolysis, motility, and resistance to antibiotics are encoded on the chromosome [3].

*B. anthracis* is a highly monophyletic clade, and isolates are differentiated by determination of single nucleotide polymorphisms (SNPs) and variable number of tandem repeats (VNTRs) [10], [11]. The pathogen is able to cause edema and cell death by a tripartite toxin consisting of the protective antigen, the edema factor, and the lethal factor [12]. The production of a polyglutamic acid capsule allows the organism to escape the immune system [13]. The virulence factors are encoded on the toxin plasmid, pXO1 [7], and the capsule plasmid, pXO2 [14]. Although sequences of pXO1 and to a lesser extent of pXO2 are widely distributed among strains of the *B. cereus* group [15], [16], the presence of plasmids encoding the toxin and capsule genes occurs only rarely.

Here we present the complete genome sequence of a *Bacillus* isolate which induced lethal anthrax in chimpanzee “Léo” in the rainforest of the Taï National Park, Côte d'Ivoire (CI) [17]. The strain belongs to a collection of genetically closely related bacteria, isolated in 2001 and 2002 from deceased wild chimpanzees living in this rain forest area (CI isolates). Pathological and histological examination of “Léo's” body revealed hemorrhages in nearly all inner organs, particularly in the intestines and lungs, and the lungs were also characterized by edema and emphysema. Microscopic examination revealed Gram-positve, rod-shaped bacteria located intra- and extravascularly in all tissues examined – spleen, liver, lung, lymph nodes, intestines – suggesting an acute bacterial infection as the cause of death [17]. Real time PCR [18] confirmed the presence of *B. anthracis*-specific markers in DNA isolated from different organ samples [17]. In 2004, related strains (CA isolates) were obtained from three chimpanzees and one gorilla that had died in the Dja Reserve, Cameroon (CA) [19], [20].

All these West and Central African strains tentatively grouped as *B. anthracis*-like isolates harbor pXO1- and pXO2-like sequences [17], [19] and share plasmid encoded features of the classic *B. anthracis* strains, like toxin and capsule production [21]. However, the isolates differ from *B. anthracis* in important microbiological features, a) they are motile, b) resistant to the γ-phage, and c) some isolates are also resistant to penicillin G [21]. Multilocus sequence typing [21]–[23] revealed a close relationship with *B. anthracis* and with two atypically virulent isolates of the *B. cereus* group: *B. thuringiensis* serovar konkukian strain 97-27 which was isolated from a case of severe human tissue necrosis and shown to be pathogenic in immonosuppressed mice [1], [24], [25], and *B. cereus* E33L which was isolated from a dead zebra suspected to have died of anthrax, but it remains unclear if it was the cause of death [26].

For the first time we present the complete genome sequence of a *Bacillus* isolate that apparently causes anthrax and possesses both virulence plasmids of *B. anthracis*, but exhibits a chromosomal background that points to a non-*B. anthracis* member of the *B. cereus* group, e. g. *B. cereus* or *B. thuringiensis*.

## Results and Discussion

### General genome features

**Figure 1:**
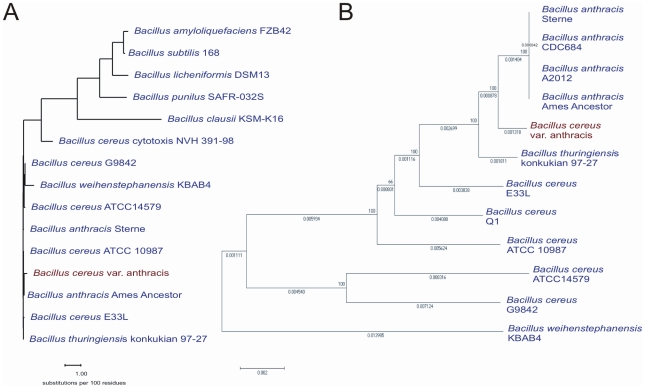
Phylogenetic analysis of “*B. cereus* var. anthracis” strain CI. (A) Phylogenetic characterization based on 16S rRNA genes. (B) Phylogenetic analysis based on multilocus sequence typing (MLST) of the *B. cereus* group [22].

The genome of “*B. cereus* variety anthracis” (Bc var. anth.) strain CI consists of four replicons, a bacterial chromosome and three plasmids encoding together 5696 protein and 162 RNA genes including 11 rRNA operons, 102 tRNA genes and 30 ncRNA genes (Table 1 and Table S1). According to the typing scheme of Sacchi et al. [27], the CI strain possesses the 16S rRNA gene type 6 like classic *B. anthracis*. The chromosome with its size of 5,488,191 bp is larger than the so far sequenced *B. anthracis* chromosomes. A phylogenetic analysis based on 16S rDNA sequences (Figure 1A) confirmed an almost complete correspondence of all *B. cereus* sensu lato strains (except the cytotoxis NVM strain). Multilocus sequence typing (MLST), however, showed that the Bc var. anth. strain CI does not cluster with the classic *B. anthracis* strains but can be grouped between them and *B. thuringiensis* serovar konkukian strain 97-27 (Figure 1B and [21]). The chromosomal background distinguishes the new isolate from typical *B. anthracis* strains and groups it as a new member of the *B. cereus* group. Most importantly, the isolate lacks the four *B. anthracis*-specific prophage regions [19], [28] and the nonsense mutation in the gene encoding the regulator PlcR [21], [29]. Bc var. anth. strain CI harbors the three plasmids pCI-XO1, pCI-XO2 and pCI-14.

**Table 1:** General genome features of bacilli from the *B. cereus* group.

Species	replicon	Size	G+C content	protein genes	% protein coding	rRNA cluster	tRNA genes	Reference
“*B. cereus* var. anthracis” CI	chromosome	5,488,191	35	5,353	80	11	102	this work
	pCI-XO1	181,907	33	214	77	-	-	
	pCI-XO2	94,469	33	111	76	-	-	
	pCI-14	14,219	38	18	65	-	-	
*B. anthracis* Ames Ancestor	chromosome	5,227,419	35	5,309	80	11	95	[87]
	pX01	181,677	32	177	62	-	-	
	pX02	94,830	33	98	63	-	-	
*B. anthracis* A2012	chromosome	5,093,554	35	5,544	81	n. d.*	n. d.	[44]
	pX01	181,677	32	204	71	-	-	
	pX02	96,829	33	104	68	-	-	
*B. anthracis* str. CDC684	chromosome	5,230,115	35	5,579	84	11	98	Dodson et al., 2009, direct submission, unpublished
	pXO1	181,773	32	206	75	-	-	
	pXO2	94,875	33	117	76	-	-	
*B. anthracis* str. Sterne	chromosome	5,228,663	35	5,281	83	11	95	Brettin et al., 2004, direct submission, unpublished
*B. cereus* G9241	chromosome	5,934,942	35	6,147	80	n. d.	n. d.	[42]; unfinished sequence
	pBClin29	29,866	35	n. d.	n. d.	-	-	
	pBCXO1	190,861	32	174	58	-	-	[42]; complete sequence
	pBC210	209,385	31	201	64	-	-	
*B. cereus* E33L	chromosome	5,300,915	35	5,134	85	13	96	[26]; JGI finishing team 2004, direct submission, unpublished
	pZK467	466,370	33	430	66	-	-	
	pZK5	5,108	30	5	65	-	-	
	pZK54	53,501	31	54	66	-	-	
	pZK8	8,191	31	8	56	-	-	
	pZK9	9,150	31	10	62	-	-	
*B. cereus* ATCC 14579	chromosome	5,411,809	35	5,476	80	13	108	[37]
	pBClin15	15,274	38	21	87	-	-	
*B. cereus* ATCC 10987	chromosome	5,224,283	35	5,603	84	12	97	[88]
	pBc10987	208,369	33	241	81	-	-	
*B. thuringiensis* serovar konkukian str. 97-27	chromosome	5,237,682	35	5,117	83	13	105	[26]; JGI finishing team 2004, direct submission, unpublished
	pBT9727	77,112	32	80	81	-	-	
*B. thuringiensis* str. Al Hakam	chromosome	5,257,091	35	4,736	82	14	104	[32]
	pALH1	55,939	36	62	73	-	-	
*B. weihenstephanensis* KBAB4	chromosome	5,602,503	35	5,532	81	n. d.	97	Lapidus et al., 2006, direct submission, unpublished

*n. d., no data.

The sequences described in this article are available at GenBank under accession numbers CP001746–CP001749.

### Identification of the “*B. cereus* var. anthracis” strain CI core and pan genome

**Figure 2:**
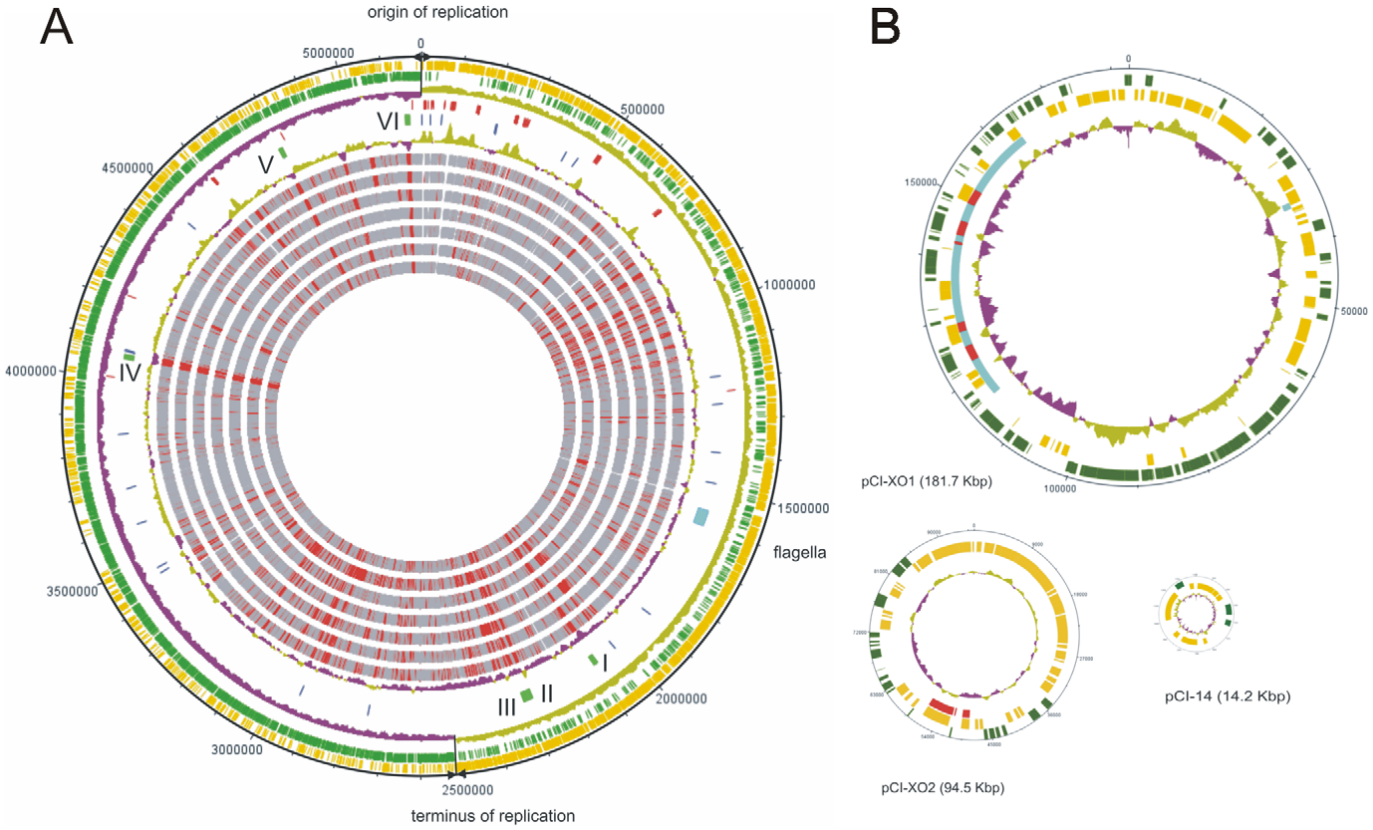
Circular maps of “*B. cereus* var. anthracis” strain CI chromosome and plasmids. (A) Circular map of Bc var. anth. CI chromosome in comparison with chromosomes of the *B. cereus* group. The map is oriented with the origin of replication on top, the direction of replication is depicted by arrowheads. The rings display from outside to the center a) ORFs, clockwise transcribed genes in gold, counterclockwise in green, b) GC-skew c) stable RNAs genes in red d) genomic islands in green, the flagella locus in light blue and repetitive elements in blue, e) GC-content, f)–l) BiBlast comparisons of strain CI with f) *B. anthracis* Ames Ancestor, g) *B. anthracis* Ames, h) *B. anthracis* Sterne, i) *B. cereus* ATCC 10987, j) *B. cereus* E33L, k) *B. thuringiensis* serovar konkukian strain 97-27, and l) *B. weihenstephanensis* strain KBA4. Shared genes are displayed in grey, missing genes in red, white regions refer to regions of Bc var. anth. strain CI that do not code for proteins. Known genomic islands are indicated by roman numbers. (B) Circular maps of the Bc var. anth. CI plasmids pCI-XO1, pCI-XO2 and pCI-14, the sizes of the circles are correlated to relative size of the plasmids. Clockwise transcribed genes are depicted in gold, counter clockwise transcribed genes in green. The inner ring displays the GC-content. Invertible elements A and B in pCI-XO1 are marked in light blue, virulence correlated genes in element B are marked red. Genes for capsule synthesis in pCI-XO2 are depicted in red.

The chromosome sequence of Bc var. anth. strain CI shares synteny over the whole length with the chromosomes of all strains of the *B. cereus* sensu lato group including the classic *B. anthracis* strains. The organization of the conserved parts of the chromosomal backbone shows a remarkably conserved structured mosaic (Figure 2A). A genome wide BiBlast comparison of Bc var. anth. strain CI with all known *Bacillus* genome sequences available at the time of analysis revealed a set of approximately 4000 (∼75% of the genes encoded per genome) orthologous genes shared by all *B. cereus* sensu lato strains with the exception of the untypical small genome of *B. cereus* subspecies *cytotoxis* NVH 391/98 [30], representing a core genome of the *B. cereus* sensu lato group (Figure S1A and B, Table S2). Bc var. anth. strain CI shares most orthologous proteins with *B. cereus* E33L (4229 orthologues) and *B. thuringiensis* serovar konkukian strain 97-27 (4180 orthologues) [1], [24], [25]. In contrast, only 4114 orthologous proteins are shared with *B. anthracis* strain Ames. If the genomes of the *B. subtilis* group are included in the analysis the number of orthologous proteins decreases to approximately 2300 genes which may represent the core genome of the genus *Bacillus* (Figure S1C).

### Genomic islands of “*B. cereus* var. anthracis” strain CI

A selected set of seven strains, four *B. anthracis*, two *B. cereus*, *B. thuringiensis* serovar konkukian and *B. weihenstephanensis* KBAB4 from the BiBlast analysis are depicted in Figure 2A. Several features are apparent. The majority of strain specific genes are located in the regions surrounding the terminus of replication. Twelve genomic regions have been identified in Bc var. anth. strain CI which encode genes absent in some or all of the compared strains and which show a clear GC-content deviation as compared to their genomic environment. Six of those regions represent islands of 12 kbp or more in size (Table 2) and are co-localized with genes correlated to mobile genomic elements i. e. integrases, recombinases and transposases. These regions might therefore be considered as strain specific genomic islands probably acquired by horizontal gene transfer [31]. The islands I, II, IV and VI were unique to Bc var. anth. strain CI (at the time of analysis). For island V a corresponding region has been found in *B. cereus* AH820, and several ORFs are distributed among the *B. cereus* group. Island III has been assigned as prophage based on the similarities to a prophage of *B. thuringiensis* Al Hakam [32]. The islands II and III are located close to each other and are separated by an insertion which is found in many *B. cereus* sensu lato strains. The majority of genes located within the genomic islands of Bc var. anth. strain CI encode proteins of unknown functions. In cases of the islands where an annotation was possible the encoded functions are often found in genomic islands [33] such as phage specific genes, a type I restriction modification system, and a transport system. The finding of defined islands within a highly syntenic chromosomal backbone supports the idea of a conserved genomic mosaic structure as described by Han et al. [26].

**Table 2:** “*B. cereus* var. anthracis” strain CI regions larger than 12 kbp and plasmid pCI-14.

Island	I	II	III	IV	V	VI	plasmid pCI-14
Genome position	2076648–2089560	2283231–2291389	2300576–2312524	4061541–4081762	4789830–4801917	5154639–5165848	
Size	13 kbp	12 kbp	13 kbp	22 kbp	12.5 kbp	12.5 kbp	14.2 kbp
Gene fragments tested in PCR*	BACI_c22180 (638 bp)	BACI_c24230 (535 bp)	BACI_c24450 (300 bp)	BACI_c43090 (745 bp)	BACI_c51040 (327 bp)	BACI_c54520 (604 bp)	BMA_pCI1400090 (448 bp)
	BACI_c22220 (677 bp)	BACI_c24340 (619 bp)	BACI_c24500 (438 bp)	BACI_c43150 (748 bp)	BACI_c51070 (354 bp)	BACI_c54560 (756 bp)	BACI_pCI1400190 (445 bp)
			BACI_c24550 (445 bp)	BACI_c43220 (426 bp)			
Biological function	unknown (glycogen branching enzyme; camelysin-like protein; hypothetical proteins)	unknown (hypothetical proteins)	putative prophage	type I restriction modification system; hypothetical proteins	transport proteins	unknown (putative ATPase; hypothetical proteins)	unknown (hypothetical proteins)

*The amplicon size is given in brackets.

The presence of genomic islands I to VI and plasmid pCI-14 in strains of the *B. cereus* group was investigated by PCR analysis (Table 2). For each region, two or three gene fragments were amplified. The analysis included 62 representative strains of *B. anthracis* comprising all six MLVA clusters except B2 [11] and deriving from Europe, Asia, Africa and unknown origins. In addition, 46 non-*B. anthracis* strains of the *B. cereus* group (16 *B. cereus*, 8 *B. thuringiensis*, one *B. mycoides*, one *B. weihenstephanensis*, 20 further strains with unclear species affiliation) were tested which represented all clades and lineages described by Priest et al. [22], including strains acquired from strain collections and all strains characterized previously [34]. The sequences derived from island III (putative prophage) were widely distributed, and singular fragments or all three fragments together were detected in a large number of strains. The fragment of BACI_c24450 (putative phage protein) was amplified in almost all *B. anthracis* strains and in 11 non-*B. anthracis* strains. The sequence fragment of BACI_c24230 (island II, hypothetical protein) was amplified in 4 non-*B. anthracis* strains of the *B. cereus* group. All other sequences tested were specific for Bc var. anth. strain CI. The distribution of the genomic islands within this variety of related strains, which does not follow the dendrograms derived by MLST, supports the hypothesis that the bacteria of the *B. cereus* group share a common pan genome of which parts can be exchanged by horizontal gene transfer. Especially the encoded prophages are therefore widely distributed within the *B. cereus* group of strains and might thereby represent a way of horizontal gene transfer.

### Island IV is an intervening sequence in the gene for sporulation factor σ^K^

**Figure 3:**
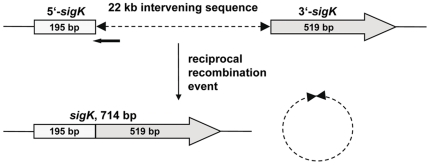
Organization of the *sigK* locus in “*B. cereus* var. anthracis” strain CI. The disrupted *sigK* gene is shown on the top. Shaded rectangles/arrows represent the 5′ and 3′ fragments of the disrupted gene. The intervening sequence is indicated by a dashed line, and the position and orientation of the recombinase gene are indicated by a black arrow. An intact *sigK* gene and a circularized molecule comprising the excised intervening sequence (bottom) are generated by a proposed reciprocal recombination event.

In *B. subtilis*, the *sigK* gene encoding the late sporulation factor σ^K^ is interrupted by a 48 kbp prophage-like element. At an intermediate stage of sporulation, the two *sigK* gene fragments are joined in frame by site-specific recombination. The recombination event is reciprocal and the intervening DNA is circularized when it is excised from the chromosome. This event does not need to be reversible because the mother cell and its chromosome are discarded after sporulation [35], [36]. The 22 kbp sequence of island IV (Table 2, BACI_c43080-BACI_c43240) is lying in the *sigK* gene of the Bc var. anth. strain CI (Figure 3). The insertion site is different from that in *B. subtilis* and the homology of the encoded proteins does not point to a putative prophage. The function of the majority of proteins is up to now unknown. However, a type I restriction modification system (R subunit: BACI_c43130, S subunit: BACI_c43150, M subunit: BACI_c43160) is encoded that is highly similar to corresponding proteins of *Geobacillus kaustophilus* and other Gram-positive bacteria but absent from bacteria of the *B. cereus* group. Type I restriction modification systems were found in *B. cereus* ATCC 14579 and ATCC 10987, but not in *B. anthracis* [37], and they occur only rarely in the *B. cereus* group. A gene for a site-specific recombinase that has 53% similarity to the *spoIVCA* recombinase gene of the *B. subtilis* intervening sequence [38] is situated directly downstream and in opposite orientation of the 5′ fragment of the *sigK* gene. Since “*B. cereus* var. anthracis” is able to sporulate efficiently, we assume that the intervening sequence is excised in the mother cell by a reciprocal recombination event similar to that described for *B. subtilis* [36] and *Clostridium difficile* [39]. The DNA rearrangement and sporulation kinetics are currently investigated. To our knowledge, this is the first description of an intervening sequence in the *sigK* gene of an isolate from the *B. cereus* group.

### Comparative genomics of the plasmids

The different lifestyles of the species of the *B. cereus* sensu lato group are largely defined by differences in plasmid-encoded features [40]. The pathogenic potential of the species *B. anthracis* is defined by the two plasmids pXO1 and pXO2, which encode the tripartite toxin and the poly-γ-d-glutamic acid capsule, respectively. *B. thuringiensis* isolates harbor plasmids that encode the insecticidal crystal proteins (Bt toxin). The *B. cereus* sensu stricto plasmid profile is extremely variable. The general features of the Bc var. anth. strain CI plasmids sequenced in the present study and those previously sequenced are outlined in Table 1. The *B. cereus* group plasmids range in size from ∼5 to 466 kb and can be divided into three groups. The first group includes pXO1-like plasmids that share a conserved core region which contains genes that are thought to be involved in plasmid replication and maintenance [40]. This group is comprised of pXO1 (*B. anthracis* strains), pBCXO1 (*B. cereus* G9241), pBc10987 (*B. cereus* ATCC 10987) and some plasmids derived from periodontal and emetic *B. cereus* isolates. The second group of plasmids includes pXO2 (*B. anthracis* strains), pBT9727 (*B. thuringiensis* serovar konkukian str. 97-27) and pAW63 (*B. thuringiensis* serovar kurstaki str. HD73) [41]. These pXO2-like plasmids share a common backbone including genes involved in replication and putative conjugative functions. The second group also comprises pBC210 (*B. cereus* G9241), pE33L466 and pE33L54 (*B. cereus* E33L) which share characteristics with pXO2 [1], [40]. Plasmid pBC210 encodes a polysaccharide capsule biosynthesis cluster [42], whereas no virulence-related functions were identified on the two large plasmids of *B. cereus* E33L [26]. “*B. cereus* var. anthracis” strain CI harbors three plasmids pCI-XO1 (181,907 bp), pCI-XO2 (94,469 bp) and pCI-14 (14,219 bp) (Figure 2B). The plasmids pCI-XO1 and pCI-XO2 fit perfectly to the groups one and two whereas pCI-14 belongs to the third group of *B. cereus* plasmids which consists of a series of smaller cryptic plasmids [40].

Comparative sequence analysis revealed that the plasmids pCI-XO1 and pCI-XO2 are highly syntenic and show 99% up to 100% identity to the plasmids pXO1 and pXO2 of *B. anthracis*. Figure S2A–D shows the results of the comparison using the whole genome alignment tool Mauve [43]. Apart from a small number of SNPs, VNTRs and single nucleotide repeats, no large insertions or deletions have been found, which confirms previous observations on this group of *B. cereus* plasmids [44]. Differences within the coding regions were not identified. The genetic variability between pCI-XO1 and other pXO1 plasmids of *B. anthracis* is not larger than the variability between the plasmids of *B. anthracis* sensu stricto (Figure S3A), and the same is true for pCI-XO2 (Figure S3B and C). The third plasmid pCI-14 was found exclusively in the isolates from chimpanzee “Léo”, not in the other two chimpanzee isolates from Côte d'Ivoire that were analyzed and in none of the isolates from Cameroon. We did not find significant similarity to any known nucleotide or protein sequences in the public sequence databases at the time of analysis, thus the function of the plasmid remains unclear. However, to our best knowledge there are no reports about any *B. anthracis* isolates harboring a third plasmid in addition to the virulence plasmids. Presence of additional plasmids is a feature thought to be characteristic of non-*B. anthracis* strains of the *B. cereus* group [40].

There are other examples of atypically virulent strains causing anthrax-like symptoms with plasmid-encoded virulence factors. *B. cereus* G9241 harbors a plasmid very similar to pXO1 (pBCXO1) and a second plasmid (pBC210) encoding a polysaccharide capsule [42]. Another strain (*B. cereus* 03BB102) that was recently sequenced harbors a plasmid (p03BB102_179) that contains both the anthrax toxin and capsule biosynthesis genes [45]. It is a known fact that pXO1- or pXO2-like plasmids or single plasmid-encoded genes can be acquired by horizontal gene transfer [41], [46]–[49], but Bc var. anth. strain CI is the first isolate in which both *B. anthracis* virulence plasmids are present in a non-*B. anthracis* chromosomal background.

### Plasmid- and chromosome-encoded virulence factors

As expected, the pXO1- and pXO2-encoded toxin components, capsule biosynthesis proteins and regulatory proteins are present in the “*B. cereus* var. anthracis” strain CI. Under inducing conditions (LB broth with 0.8% bicarbonate in a 5% CO_2_ atmosphere), protective antigen (PA), lethal factor (LF) and edema factor (EF) were synthesized [21] and immunostaining of bacteria with the monoclonal antibody F26G3 [50] confirmed the production of an anthrax-like capsule (data not shown). Compared to *B. anthracis* Ames Ancestor, PA, EF and LF contain three, four, and eight amino acid exchanges, respectively. Seven of the eight amino acid exchanges of LF and one of the four exchanges in EF result in related amino acids. The transcriptional regulator AtxA [51] differs by one amino acid from the protein of *B. anthracis* Ames Ancestor. Interestingly, the CI strain encodes new variants of PA [19], [45], EF and the PagR regulator [52] that are also found on the pXO1-like plasmid pBCXO1 of *B. cereus* G9241. The *bslA* gene which encodes a putative adhesin [53] contains the same frameshift mutation in pCI-XO1 and in pBCXO1.

The “*B. cereus* var. anthracis” strain CI possesses several known chromosomally encoded virulence factors of the *B. cereus* group (Table S3) like hemolysins, non-hemolytic enterotoxins and phospholipases [54]. Like in *B. anthracis* and *B. cereus* E33L, the complete 17.7-kbp insertion comprising the *gerI/hbl* operon is lacking in the CI strain [26]. Some plasmid-encoded virulence factors (not shown in the table) like the crystal proteins (δ-endotoxins) of *B. thuringiensis* [8] and the emetic toxin of emetic strains of *B. cereus* [9] were also absent from Bc var. anth. strain CI. Internalin proteins located at the bacterial surface are known to interact with host cells via specific protein receptors [55]. Two putative internalins were detected in the CI strain genome and were found at comparable genome positions as in other *B. cereus* group chromosomes. BACI_c13660 exhibits high similarity (more than 90% identity) to proteins from other strains of the *B. cereus* group, but like in *B. anthracis* it is truncated at the N-terminus due to a frameshift mutation. BACI_c05600, however, is only weakly/partially homologous to other internalin proteins found at the corresponding genome position in other strains (Table S4).

### The PlcR regulon in “*B. cereus* var. anthracis” strain CI

Recent analyses showed that the pleiotropic regulator PlcR regulates the expression of 45 genes, including many virulence-related genes, in the reference strain *B. cereus* ATCC 14579, and a similar result can be expected for other strains of the *B. cereus* group [56]. In *B. anthracis*, the regulator is not functional due to a nonsense mutation in the *plcR* gene [29]. Despite the fact that most of the potential members of the PlcR-regulon as described by Ivanova et al. [37] are present in Bc var. anth. strain CI and that the corresponding transcription units are encoded downstream of *plcR* boxes our results so far indicate that PlcR is also not functional. The PlcR-regulated phosphatidylinositol-specific phospholipase C protein is inactive in several tests: i) colonies did not exhibit a color change on Cereus Ident agar [21]; ii) no PCR-product was obtained by reverse transcriptase PCR with RNA from Bc var. anth. strain CI; and iii) in western blot, culture supernatants did not react with a phospholipase C specific antibody. In all experiments, the type strain *B. cereus* DSM 31 (corresponding to ATCC 14579) reacted positive as expected (data not shown). Further reverse transcriptase PCR analyses were conducted to detect the mRNA for PlcR-regulated genes. However, expression of the genes for cereolysin O (*clo*), phosphatidylcholine specific phospholipase C (*plcB*) and a serine protease (*sfp*) (Table S3) was comparable to *B. anthracis* and either completely abolished or substantially weaker compared to the *B. cereus* DSM 31 control strain. We assume that PlcR is not active in Bc var. anth. strain CI because its C-terminus that is important for interaction with the PapR cell-cell signaling peptide is altered [57]. A frameshift mutation (insertion of an A-residue) near the stop codon results in a C-terminus of the protein that is slightly altered and four amino acids longer than usual: —SIIKKNEEMKRT compared to —SIIKRMKK in *B. thuringiensis* serovar konkukian. In addition, the gene for the OppA protein of the OppABCDF transport system that is responsible for reimport of PapR into the cell [58] contains a frameshift mutation in Bc var. anth. CI. Interestingly, identical frameshift mutations in *plcR* and *oppA* were detected in all strains from Côte d'Ivoire and Cameroon that were analysed, suggesting that they represent a clonally derived lineage.

### Motility of “*B. cereus* var. anthracis” strain CI

**Figure 4:**
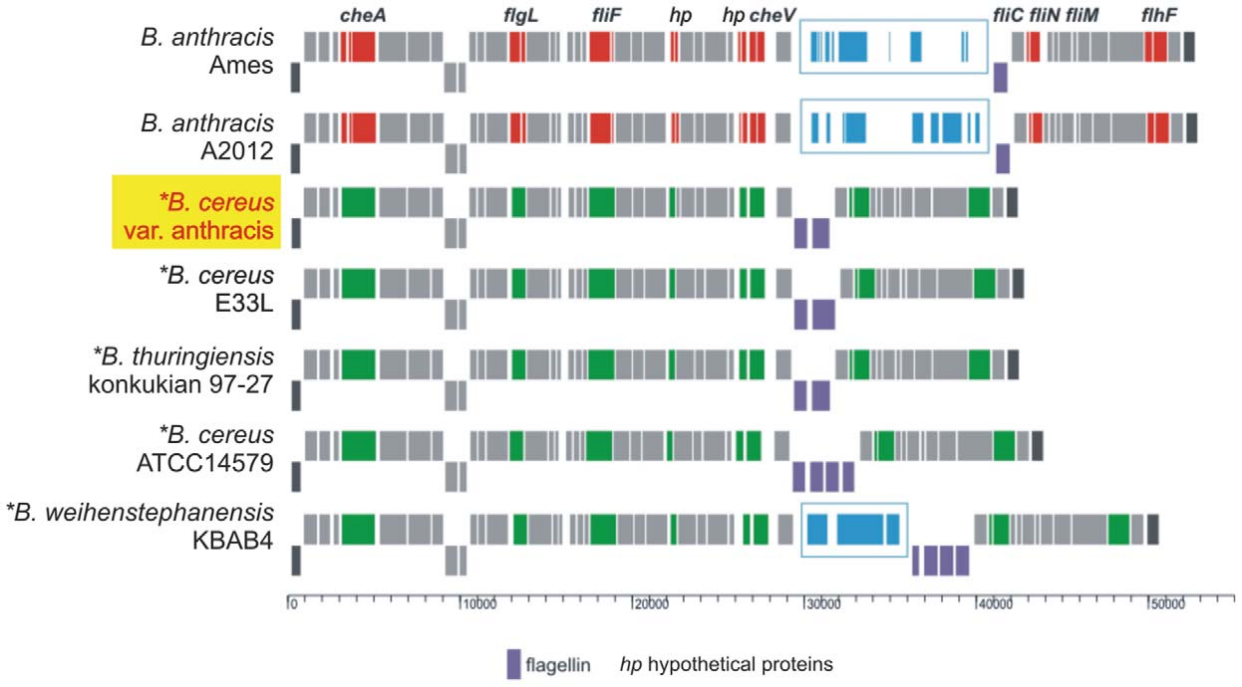
Comparison of the flagella gene loci encoding flagella genes in strains of the *B. cereus* group. Motile strains are marked with an asterisk. Nonfunctional genes are depicted in red, corresponding functional genes in green, intact corresponding genes shared by all strains are grey. The essential flagellin genes have been marked purple and inserted gene blocks in blue. “*B. cereus* var. anthracis” CI contains apparently a fully functional motility locus like strains *B. cereus* E33L and *B. thuringiensis* konkukian 97-27. *B. cereus* ATCC 14579 and *B. weihenstephanensis* KBA4 contain a duplication of the flagellin genes. The insertion of additional sequences and accordingly the duplication of genes occur in corresponding regions of the motility locus.

In contrast to *B. anthracis*, bacteria of the Bc var. anth. strain CI exhibited motility. A detailed comparison of the flagella biosynthesis cluster of strain CI with the corresponding gene clusters of two *B. anthracis* strains and four *B. cereus* sensu lato strains revealed a fully functional gene cluster (Figure 4). Ten motility- and chemotaxis-associated genes that contain frameshift mutations in *B. anthracis* Ames Ancestor are intact in the Bc var. anth. strain CI: *motA* (BACI_c16760), *cheA* (BACI_c16790), *flgL* (BACI_c16880), *fliF* (BACI_c16950), BA1682 (BACI_c16990), BA1688/BA1689 (BACI_c17050), *cheV* (BACI_c17060), *fliN* (BACI_c17120), *fliM* (BACI_c17130), and *flhH* (BACI_c17210). Like *B. thuringiensis* serovar konkukian and *B. cereus* E33L, the CI strain possesses two flagellin genes *fliC1* and *fliC2* (BACI_c17090 and BACI_c17100), whereas *B. anthracis* Ames has only one and *B. cereus* ATCC 14579 has four flagellin genes. The varying numbers of flagellin genes and the insertion of three different additional sets of genes at the flagellin locus in the *B. anthracis* strains and *B. weihenstephanensis* might indicate an evolutionary hotspot.

Older studies suggested that motility genes are also regulated by the PlcR regulon. Expression of flagellin genes was downregulated threefold in a *plcR* mutant [59], and PlcR boxes were found in the promoter regions of genes related to motility and chemotaxis [37]. However, in the recent publication by Gohar et al. [56] where a variety of methods was used to determine the genes regulated by PlcR, no motility genes were identified. Therefore, motility of Bc var. anth. CI can be explained despite the putative inactivity of PlcR.

### Protein secretion systems

**Figure 5:**
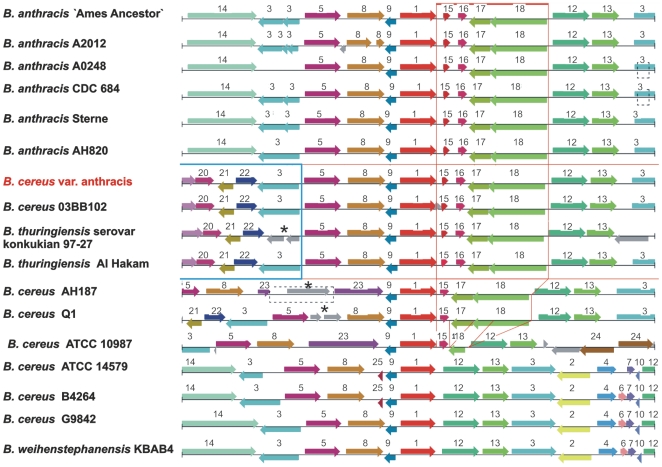
Comparative genome alignment of the *secA2* locus in members of the *B. cereus* sensu lato group. The numbers indicate ORFs 18: *secA2*, 15–17: conserved hypothetical proteins, 1: sulfate transporter, 12/13: *csaA*/*csaB* polysaccharide synthase subunits. * mobile genetic elements.

The secretion of proteins is crucial for the pathogenic life style within the *B. cereus* group. “*B. cereus* var. anthracis” strain CI contains apparently two *sec*-type secretion systems. One system is fully orthologous to the *B. subtilis* system for the secretion of unfolded proteins [60]. The second system is orthologous to the so called *secA2* system from *B. anthracis* and other Gram-positive pathogens. The *secA2* secretion system is thought to secrete a specific subset of proteins associated with pathogenicity [60]–[62]. A comparative genome alignment revealed that Bc var. anth. strain CI contains a *secA2* locus which is organized exactly like in *B. anthracis* and closely related *B. cereus* group strains (Figure 5). Upstream of this locus the CI genome is organized like the *B. thuringiensis* strains and the majority of *B. cereus* strains. Interestingly, the strain CI genes are integrated in the corresponding core genome position of their orthologous counterparts in the *B. anthracis* strains respectively in the genome of *B. cereus* AH187. A phylogenetic tree of the SecA2 protein sequences revealed a close relationship of the proteins (identities around 99%) except for the *B. cereus* cytotoxis strain NVH391-98 (identity 86%) and the *B. thuringiensis* serovar konkukian strain 97-27 (identity 81%) (Figure S4A). Comparison of the *secA2* secreted S-layer proteins Sap and EA1 encoded downstream of the *secA2* locus indicated that both proteins from Bc var. anth. strain CI cluster exclusively with the *B. cereus* variants and not with the proteins encoded by *B. anthracis* strains (Figure S4B). Interestingly, *B. thuringiensis* serovar konkukian does not possess homologs of the S-layer proteins Sap and EA1, but encodes two different S-layer proteins at the corresponding genome position that might have been acquired by horizontal gene transfer.

### Evolution of genes

The MLST method is based upon phylogenetic comparison of conserved housekeeping genes and is therefore well suited to follow the path of evolution of a given set of genes by point mutations [63], [64]. Following MLST based on the genes classically used for strains of the *B. cereus* group [22], in which recombination events occur less often than point mutations, the CI strain is a member of clade 1 comprising *B. anthracis* and mainly *B. cereus* strains (Figure 1B and [21]). However, it was found that gene acquisition from strains clustering outside the known MLST database is common among clade 1 strains [65]. Consequently the phylogenetic analysis on the S-layer proteins confirmed the intermediate position of strain CI (Figure S4B) between *B. cereus* E33L on one side and all classic *B. anthracis* strains on the other side. These results show the importance of the gene selection for the clustering of a strain by MLST. BiBlast, used for general genome comparison (Figure S1), identified common orthologous proteins within all bacilli genomes. The knowledge of orthologous genes shared by *B. cereus* genomes identified the group of genes which evolve by point mutations and are thus suitable for phylogenetic analysis.

### Evolution of genomes and epidemiology of *B. anthracis* strains

The genomes of the *B. cereus* group exhibit a conserved mosaic structure (Figure 2A and [26]). Singular genes and operons of Bc var. anth. CI encoding diverse virulence factors and antibiotic resistance are differently distributed between strains of the *B. cereus* group. Some virulence associated operons and their genomic environment are present in all strains, others are restricted to a small number of strains (Table S3 and [66]). Examples are the mersacidin resistance operon that until now was only found in few strains of the *B. cereus* group and in the CI strain and the *secA2* operon described above (Figures 5 and S4). Comparable genomic mosaic structures have been found in several organisms of distant phylogenetic groups [67]–[69]. These structures are usually correlated with the presence of mobile genetic elements like insertion sequence elements, phages, transposases, integrases and recombinases and represent an evidence for strain evolution by horizontal gene transfer. In addition, plasmid transfer within the *B. cereus* group is well established, and there are numerous mobility genes on pXO1 and conjugative functions on pXO2 [41], [48], [49]. *B. anthracis* plasmids are not self-transmissible, but both pXO1 and pXO2 could be transferred from *B. anthracis* to plasmid-cured *B. anthracis* or *B. cereus* recipients with the aid of a mobilizing plasmid [46], [47].

In *B. anthracis*, regulatory mechanisms link chromosomally encoded and plasmid-encoded genes. Some chromosomal genes were shown to be regulated by the plasmid-encoded regulator AtxA [70]. For example, the chromosomal S-layer genes *sap* and *eag* are regulated by AtxA in a way that only *eag* is significantly expressed under inducing conditions with CO_2_ and bicarbonate [71]. In addition, *B. anthracis* does not sporulate while growing in the blood of the host but requires the activity of the sporulation initiation pathway and Spo0A to express toxin genes [72]. One of several sporulation sensor kinase genes (BA2636) is inactivated by two different frameshift mutations in *B. anthracis* and in *B. cereus* G9241 [73]. It was proposed that acquisition of plasmid pXO1 and pathogenicity may require a dampening of sporulation regulation by mutational selection of sporulation sensor histidine kinase defects. However, no frameshift mutations were detected in the BA2636 homolog of Bc var. anth. CI, and no obvious mutations were found in the other eight potential genes for sporulation sensor histidine kinases. It is possible that regulatory systems of plasmids and chromosome are not linked in a way that is observed in classic *B. anthracis*, and one reason for that might be that the plasmids were acquired relatively recently and are not yet fully adapted to the chromosome. Further experiments will be performed to assess the linkage between chromosomally and plasmid-encoded genes.

A prerequisite for horizontal gene transfer is the direct contact (conjugation) or indirect contact (transformation or transduction) of donor and recipient strains as vegetative cell. Based on previous results, conjugation is the most probable way of plasmid transfer in the *B. cereus* group [41], [74]. In the past, it was thought that in the environment, *B. anthracis* strains primarily exist as a dormant, highly stable spore and vegetative cells are limited to the stages inside the host [6]. However, it was shown that some strains of *B. anthracis* can germinate in the rhizosphere and grow in characteristic long filaments, in which plasmid transfer was documented [75]. *B. cereus* and *B. thuringiensis* are ubiquitous soil microorganisms that are able to germinate, grow, and sporulate in the rhizosphere of plants or in soil [76], [77]. Genetic exchange resulting in a *B. cereus* group bacterium possessing the anthrax plasmids is therefore possible both during co-infection in a host or in the soil.

The new *B. anthracis* isolates have been exclusively detected in CI and CA, but may be present in other regions of Africa where they were eventually misdiagnosed using microbiological methods because they differ from classic anthrax. The ecology of the bacteria is atypical, because they were found in primates in a rain forest area, and classic anthrax is usually a disease of herbivores in the savannah [20]. “*B. cereus* var. anthracis” strain CI i) shares more orthologous genes with *B. cereus* E33L and *B. thuringiensis* serovar konkukian strain 97-27 than with any *B. anthracis* strain, ii) contains a chromosomal mutation inactivating the PlcR regulon different from all known *B. anthracis* strains, iii) contains a functional motility operon and iv) harbors pXO1 and pXO2 plasmids in the same range of variability like typical anthrax plasmids. Therefore, one might conclude that strain CI represents a *B. anthracis* subspecies endemic in rain forests that evolved recently from a motile progenitor similar to *B. cereus* E33L and *B. thuringiensis* serovar konkukian strain 97-27.

### Species concept

*B. anthracis* was named as the cause of the disease anthrax [1], [78]. In the *B. cereus* group of organisms, virulence and pathogenicity appear to be promiscuous and spread with plasmids [40]. The bacterial chromosomes of this group show a high level of synteny and very high numbers of orthologous genes are shared (Figure S1A–C and Table S2). Such a combination is not observed in any other group of comparably related bacterial genomes. Furthermore, there is evidence for a shared set of core putative virulence factors between different pathogenic and non-pathogenic members of the group (Table S3). Very few chromosomal genes or sets of genes are unique to one species. Subtle changes to regulatory networks may be responsible for the range of phenotypic traits displayed by the *B. cereus* group members. Based on the classic 16S rDNA phylogeny it is not possible to distinguish members of the *B. cereus* group [1]. Recently it was suggested to designate strains that appear to reside at the boundary between *B. cereus* and *B. anthracis* as *B. cereus/B. anthracis* sensu lato strains [79]. Based on the finding that the isolate described here represents a bacterium that possesses a chromosomal background of a non-*B. anthracis* member of the *B. cereus* group, harbors both the pXO1 and pXO2 virulence plasmids of *B. anthracis* and apparently causes anthrax, we suggest to designate this and related isolates as “*B. cereus* var. anthracis” strains CI and CA.

## Methods

### Genome Sequencing

DNA from “*B. cereus* var. anthracis” strain CI was isolated using CTAB treatment and phenol-chloroform extraction as described previously [80]. For preparation of whole shotgun libraries, DNA was fragmented to sizes between 1.5 and 3.0 kbp by appropriate mechanical shearing (Hydroshear, GeneMachines, San Carlos CA, USA). DNA fragments were separated by gel electrophoresis after end-repair and cloned using vector pCR4.1-TOPO (TOPO-TA Cloning Kit for Sequencing; Invitrogen, Karlsruhe, Germany). A total of about 45,600 plasmids were isolated using two BioRobots8000 (Qiagen, Hilden, Germany) and 71,701 sequences were automatically analyzed on 3730XL (Applied Biosystems, Darmstadt, Germany) and assembled into four replicons. PCR-based techniques on genomic DNA resulted in 3,850 reads which were taken to close remaining gaps and to ensure a minimum quality value of phred 45 on each position within the genome. PCR have been carried out with the BioXact Kit (Qiagen, Hilden, Germany) as described by the manufacturer with product depending variations according the cycling program and the amount of enzyme.

### Bioinformatics

Coding sequences (CDS) and open reading frames (ORFs) were predicted with YACOP [81] using therein the ORF-finders Glimmer, Critica and Z-curve. All CDS have been manually curated and were verified by comparison with the publicly available databases SwissProt, GenBank, ProDom, COG, and Prosite using the annotation software ERGO [82]. Complete genome comparisons were done with ACT [83] based on replicon specific nucleotide BLAST [84] and with protein based BiBlast comparisons to all known sequenced bacilli (A. Wollherr, personal communication). Phylogenetic analysis was done with the programs of the PHYLIP software suite [85] and the MEGA4 software using ClustalW multiple sequence alignment for deriving a Neighbor-Joining based tree and bootstrapping with 1000 replicants [86].

### Comparative analysis of members of the *B. cereus* group by PCR screening of selected genomic regions

Standard PCR was performed for the detection of six chromosomal genomic islands and plasmid pCI-14 among a panel of strains from the *B. cereus* group. Primers (Metabion, Martinsried, Germany) were designed complementary to sequences of the CI strain and used to amplify PCR products in the range from 300 bp to 800 bp (Table 2). The reaction volume was 25 µl with 2.5 µl 10× buffer, 0.2 mM of each dNTP, 1.5 mM MgCl_2_, 0.6 units of *Taq* polymerase (Fermentas, St. Leon-Rot, Germany), 0.2 µM of each primer and 10–50 ng of template DNA. The PCR program consisted of one step at 95°C for 5 min, followed by 35 cycles with 95°C for 30 s, 50°C for 30 s and 72°C for 45 s, and a final step at 72°C for 10 min. The primer sequences are available upon request.

## Supporting Information

Figure S1 Shared chromosomal genes identified by bidirectional BLAST (BiBlast) of “B. cereus var. anthracis” strain CI and selected chromosomes of bacilli. The colors indicate the number of shared genes with the other strain. Due to strain specific multi copy genes the numbers differ depending on the direction of the BiBlast comparison. (a) Strains “B. cereus var. anthracis” CI, B. anthracis Ames Ancestor and B. cereus E33L, (b) strains “B. cereus var. anthracis” CI, B. anthracis Ames Ancestor and B. weihenstephanensis KBA4 and (c) strains “B. cereus var. anthracis” CI, B. anthracis Ames Ancestor and B. licheniformis DSM13.
(6.33 MB TIF)

Figure S2 Whole replicon sequence alignments of known pXO1- and pXO2-like plasmids with MAUVE. The colors indicate blocks of high similarity. (a) Sequence alignments of pXO1 plasmids, two invertible regions A and B enveloped by transposases have been identified. Region A represents an IS element and region B represents a 44.5 kbp pathogenicity island encoding the anthrax related virulence factors. (b) Sequence alignment of pCI-XO1 and B. cereus G9241 plasmid pBCXO1, a pXO1-like plasmid harboring the pathogenicity island encoding the anthrax toxin. (c) Sequence alignment of pXO2 plasmids. (d) pCI-XO2 versus B. thuringiensis serovar konkukian plasmid pBT9727 lacking the pathogenicity island (PAI). Reference: Darling ACE, Mau B, Blatter FR, Perna NT (2004) Mauve: Multiple alignment of conserved genomic sequence with rearrangements. Genome Research 14: 1394–1403.
(12.32 MB TIF)

Figure S3 Evolutionary relationships of pXO1 and pXO2 plasmids. The evolutionary history was inferred using the Neighbor-Joining method [1]. The bootstrap consensus tree inferred from 500 replicates [2] is taken to represent the evolutionary history of the taxa analyzed. Branches corresponding to partitions reproduced in less than 50% bootstrap replicates are collapsed. The percentage of replicate trees in which the associated taxa clustered together in the bootstrap test (500 replicates) is shown next to the branches [2]. The tree is drawn to scale, with branch lengths in the same units as those of the evolutionary distances used to infer the phylogenetic tree. The evolutionary distances were computed using the Maximum Composite Likelihood method [3] and are in the units of the number of base substitutions per site. Codon positions included were 1st+2nd+3rd+Noncoding. All positions containing gaps and missing data were eliminated from the dataset (Complete deletion option). There were a total of 180063 positions in the final dataset. Phylogenetic analyses were conducted in MEGA4 [4]. (a) Phylogenetic tree calculated on full length alignments from pXO1 plasmids of Bc var. anth. CI and different B. anthracis strains. The invertable elements are normalized. (b) Phylogenetic tree calculated on full length alignments from pXO2 plasmids of Bc var. anth. CI and different B. anthracis strains. (c) Phylogenetic tree with pXO2 plasmids from Bc var. anth. CI, different B. anthracis strains and two related plasmids from B. thuringiensis. For plasmids of B. anthracis strains, only the strain designations are indicated. References: 1. Saitou N, Nei M (1987) The neighbor-joining method: A new method for reconstructing phylogenetic trees. Mol Biol Evol 4: 406–425. 2. Felsenstein J (1985) Confidence limits on phylogenies: An approach using the bootstrap. Evolution 39: 783–791. 3. Tamura K, Nei M, Kumar S (2004) Prospects for inferring very large phylogenies by using the neighbor-joining method. Proc Natl Acad Sci U S A 101: 11030–11035. 4. Tamura K, Dudley J, Nei M, Kumar S (2007) MEGA4: Molecular Evolutionary Genetics Analysis (MEGA) software version 4.0. Mol Biol Evol 24: 1596–1599.
(11.24 MB TIF)

Figure S4 Phylogenetic comparison of SecA2 and S-layer proteins. (a) Rooted phylogenetic tree of SecA2 proteins (b) Phylogenetic tree of S-layer proteins Sap and EA1.
(12.43 MB TIF)

Table S1 Stable RNAs and Riboswitches
(0.03 MB DOC)

Table S2 Core and Pan genome of the “B. cereus var. anthracis” strain CI genome and selected Bacillus strains.
(0.04 MB DOC)

Table S3 Presence or absence of virulence factors and regulatory proteins in “B. cereus var. anthracis” strain CI.
(0.15 MB DOC)

Table S4 Identity of internalin proteins present at comparable genome positions.
(0.03 MB DOC)
