# Supplementary figures and images for: The Genome of a Bacillus Isolate Causing Anthrax in Chimpanzees Combines Chromosomal Properties of B. cereus with B. anthracis Virulence Plasmids
Source: PLoS One. 2010 Jul 9;5(7):e10986. doi: 10.1371/journal.pone.0010986 (PMC2901330; doi:10.1371/journal.pone.0010986)

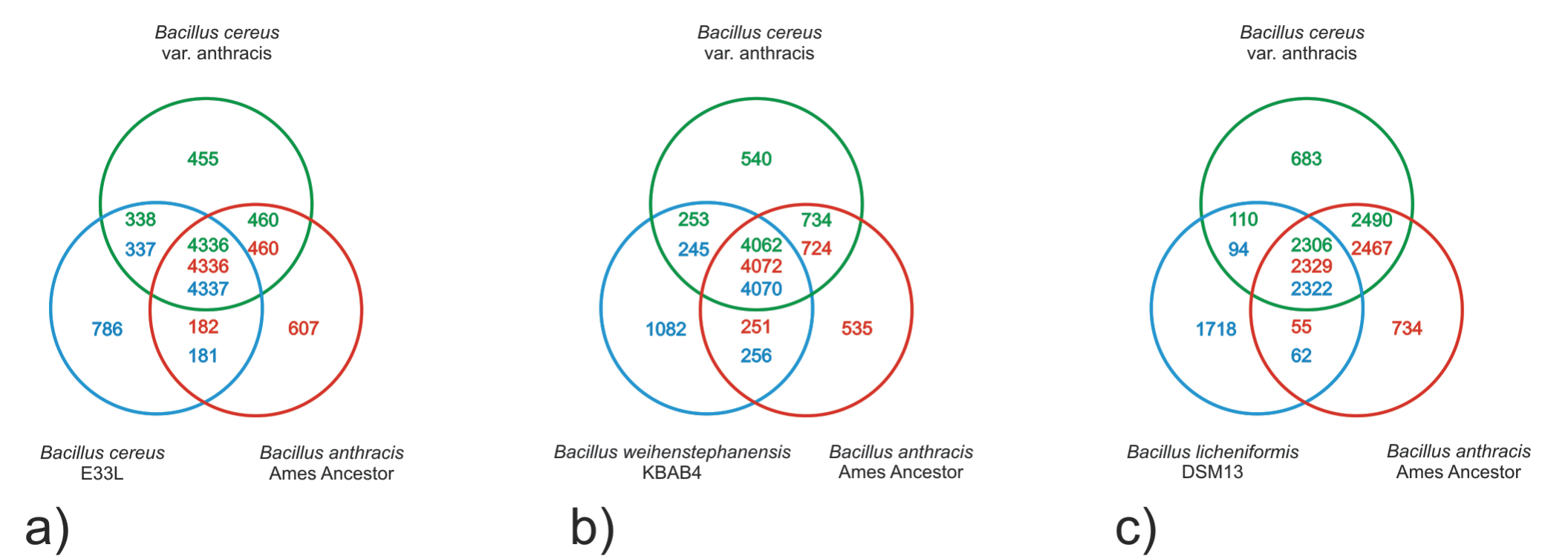

Supplement: Figure S1 — Shared chromosomal genes identified by bidirectional BLAST (BiBlast) of “B. cereus var. anthracis” strain CI and selected chromosomes of bacilli. The colors indicate the number of shared genes with the other strain. Due to strain specific multi copy genes the numbers differ depending on the direction of the BiBlast comparison. (a) Strains “B. cereus var. anthracis” CI, B. anthracis Ames Ancestor and B. cereus E33L, (b) strains “B. cereus var. anthracis” CI, B. anthracis Ames Ancestor and B. weihenstephanensis KBA4 and (c) strains “B. cereus var. anthracis” CI, B. anthracis Ames Ancestor and B. licheniformis DSM13. (6.33 MB TIF) [file pone.0010986.s001.tif]

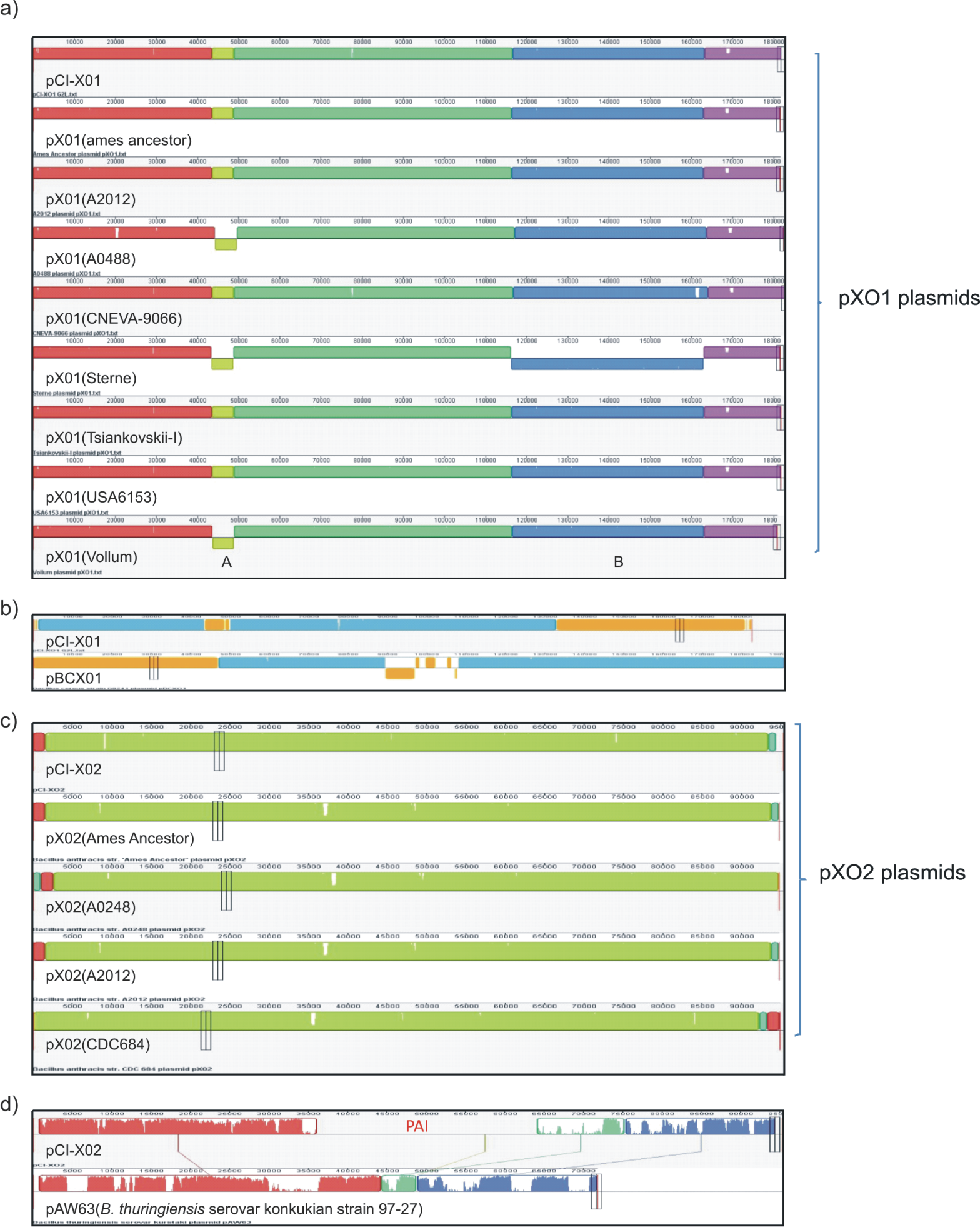

Supplement: Figure S2 — Whole replicon sequence alignments of known pXO1- and pXO2-like plasmids with MAUVE. The colors indicate blocks of high similarity. (a) Sequence alignments of pXO1 plasmids, two invertible regions A and B enveloped by transposases have been identified. Region A represents an IS element and region B represents a 44.5 kbp pathogenicity island encoding the anthrax related virulence factors. (b) Sequence alignment of pCI-XO1 and B. cereus G9241 plasmid pBCXO1, a pXO1-like plasmid harboring the pathogenicity island encoding the anthrax toxin. (c) Sequence alignment of pXO2 plasmids. (d) pCI-XO2 versus B. thuringiensis serovar konkukian plasmid pBT9727 lacking the pathogenicity island (PAI). Reference: Darling ACE, Mau B, Blatter FR, Perna NT (2004) Mauve: Multiple alignment of conserved genomic sequence with rearrangements. Genome Research 14: 1394–1403. (12.32 MB TIF) [file pone.0010986.s002.tif]

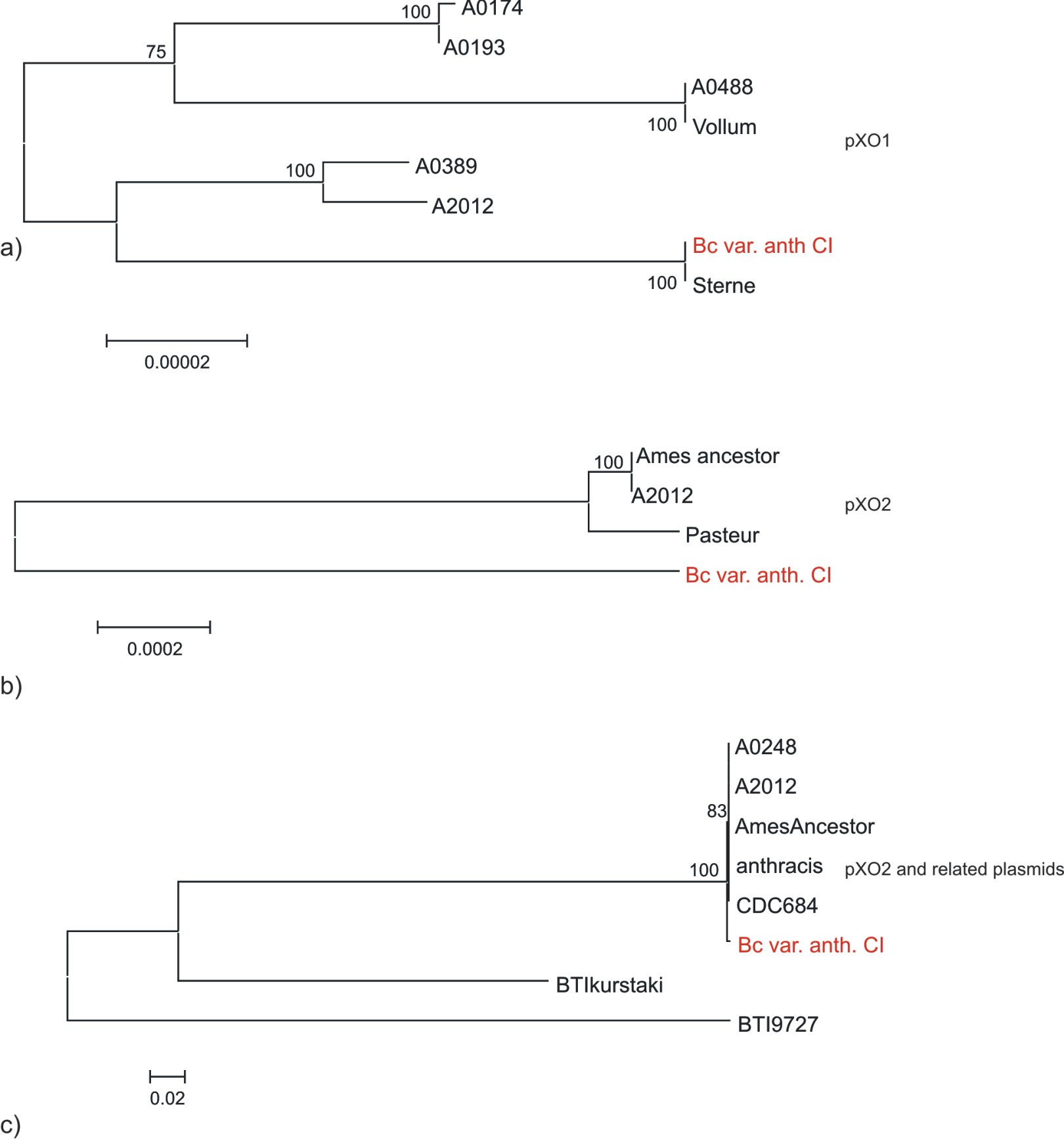

Supplement: Figure S3 — Evolutionary relationships of pXO1 and pXO2 plasmids. The evolutionary history was inferred using the Neighbor-Joining method [1]. The bootstrap consensus tree inferred from 500 replicates [2] is taken to represent the evolutionary history of the taxa analyzed. Branches corresponding to partitions reproduced in less than 50% bootstrap replicates are collapsed. The percentage of replicate trees in which the associated taxa clustered together in the bootstrap test (500 replicates) is shown next to the branches [2]. The tree is drawn to scale, with branch lengths in the same units as those of the evolutionary distances used to infer the phylogenetic tree. The evolutionary distances were computed using the Maximum Composite Likelihood method [3] and are in the units of the number of base substitutions per site. Codon positions included were 1st+2nd+3rd+Noncoding. All positions containing gaps and missing data were eliminated from the dataset (Complete deletion option). There were a total of 180063 positions in the final dataset. Phylogenetic analyses were conducted in MEGA4 [4]. (a) Phylogenetic tree calculated on full length alignments from pXO1 plasmids of Bc var. anth. CI and different B. anthracis strains. The invertable elements are normalized. (b) Phylogenetic tree calculated on full length alignments from pXO2 plasmids of Bc var. anth. CI and different B. anthracis strains. (c) Phylogenetic tree with pXO2 plasmids from Bc var. anth. CI, different B. anthracis strains and two related plasmids from B. thuringiensis. For plasmids of B. anthracis strains, only the strain designations are indicated. References: 1. Saitou N, Nei M (1987) The neighbor-joining method: A new method for reconstructing phylogenetic trees. Mol Biol Evol 4: 406–425. 2. Felsenstein J (1985) Confidence limits on phylogenies: An approach using the bootstrap. Evolution 39: 783–791. 3. Tamura K, Nei M, Kumar S (2004) Prospects for inferring very large phylogenies by using the neighbor-joining meth [file pone.0010986.s003.tif]

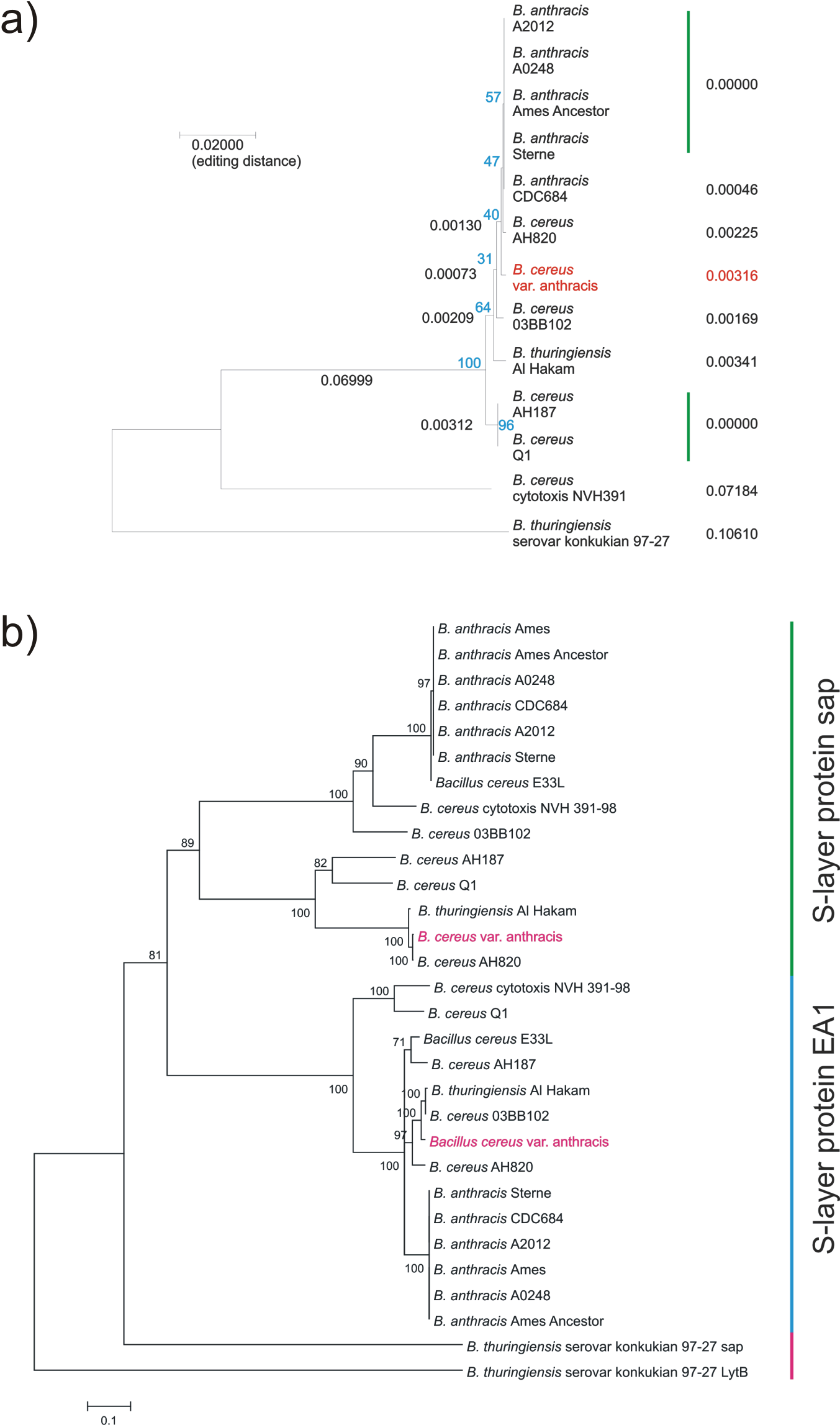

Supplement: Figure S4 — Phylogenetic comparison of SecA2 and S-layer proteins. (a) Rooted phylogenetic tree of SecA2 proteins (b) Phylogenetic tree of S-layer proteins Sap and EA1. (12.43 MB TIF) [file pone.0010986.s004.tif]
