# Supplementary material for: The Genome of a Bacillus Isolate Causing Anthrax in Chimpanzees Combines Chromosomal Properties of B. cereus with B. anthracis Virulence Plasmids
Source: PLoS One. 2010 Jul 9;5(7):e10986. doi: 10.1371/journal.pone.0010986 (PMC2901330; doi:10.1371/journal.pone.0010986)
Supplement: Table S1 — Stable RNAs and Riboswitches (0.03 MB DOC) [file pone.0010986.s005.doc]

**Table S1.** Stable RNAs and Riboswitches.

| RNA-class | chromosome | pCI-XO1 | pCI-XO2 | pCI-14 |
| --- | --- | --- | --- | --- |
| tRNA | 102 | - | - | - |
| rRNA | 33 (11 cluster) | - | - | - |
| ncRNA | 21 | 4* | 1* | 4* |
| riboswitch | 104 | 1 | 1 | - |
| tmRNA | 1 | - | - | - |

*Including ncRNAs similar to antisense RNAs known to be involved in plasmid copy number control.
