# Supplementary material for: The Genome of a Bacillus Isolate Causing Anthrax in Chimpanzees Combines Chromosomal Properties of B. cereus with B. anthracis Virulence Plasmids
Source: PLoS One. 2010 Jul 9;5(7):e10986. doi: 10.1371/journal.pone.0010986 (PMC2901330; doi:10.1371/journal.pone.0010986)
Supplement: Table S2 — Core and Pan genome of the “B. cereus var. anthracis” strain CI genome and selected Bacillus strains. (0.04 MB DOC) [file pone.0010986.s006.doc]

**Table S2.** Core and Pan genome of the “*B. cereus* var. anthracis” strain CI genome and selected *Bacillus* strains.

| Species | No of orthologues* ( ≥ 90% similarity) | No of orthologues* ( ≥ 10% similarity) |
| --- | --- | --- |
| *B. cereus* E33L | 4229 (76%) | 4628 (83%) |
| *B. thuringiensis* serovar konkukian strain 97-27 | 4180 (75%) | 4574 (82%) |
| *B. anthracis* Ames Ancestor | 4115 (74%) | 4552 (82%) |
| *B. anthracis* Ames | 4114 (74%) | 4553 (82%) |
| *B. anthracis* Sterne | 4089 (73%) | 4647 (83%) |
| *B. thuringiensis* Al Hakam | 3837 (69%) | 4305 (77%) |
| *B. cereus* ATCC 10987 | 3822 (68%) | 4354 (78%) |
| *B. cereus* ATCC 14579 | 3695 (66%) | 4310 (77%) |
| *B. weihenstephanensis* KBAB4 | 3640 (65%) | 4270 (76%) |
| *B. cereus* cytotoxis NVH 391-98 | 2010 (36%) | 3144 (56%) |
| *B. licheniformis* DSM13 | 189 (3%) | 2403 (43%) |
| *B. subtilis* 168 | 168 (3%) | 2355 (42%) |
| *B. amyloliquefaciens* FZB42 | 162 (3%) | 2315 (41%) |
| *B. pumilus* SAF-032S | 143 (3%) | 2238 (40%) |
| *B. halodurans C-125* | 101 (2.5%) | 2147 (38%) |
| *B. clausii* KSM-K16 | 72 (1%) | 2177 (39%) |

*The fraction of proteins with a BiBlast hit in relation to the total number of proteins from the compared genome is given in brackets.
