# Supplementary material for: The Genome of a Bacillus Isolate Causing Anthrax in Chimpanzees Combines Chromosomal Properties of B. cereus with B. anthracis Virulence Plasmids
Source: PLoS One. 2010 Jul 9;5(7):e10986. doi: 10.1371/journal.pone.0010986 (PMC2901330; doi:10.1371/journal.pone.0010986)
Supplement: Table S3 — Presence or absence of virulence factors and regulatory proteins in “B. cereus var. anthracis” strain CI. (0.15 MB DOC) [file pone.0010986.s007.doc]

Table S3. Presence or absence of virulence factors and regulatory proteins in “*B. cereus* var. anthracis” strain CI.

| Gene | Function of the gene | *B. anthracis* Ames Ancestor | *B. thuringiensis* konkukian 97-27 | *B. cereus* E33L | *B. cereus* ATCC14579 | *B. cereus* var. anthracis strain CI | Remarks |
| --- | --- | --- | --- | --- | --- | --- | --- |
| *hblC* | hemolytic enterotoxin BL lytic component L2 | absent | BT9727_2893 | absent | BC3104* | absent | *hblCDBA*: hemolysin operon |
| *hblD* | hemolytic enterotoxin BL binding component B | absent | BT9727_2892 | absent | BC3103* | absent |  |
| *hblA* | hemolytic enterotoxin BL binding component B | absent | BT9727_2891 | absent | BC3102* | absent |  |
| *hblB* | hemolytic enterotoxin BL binding component B’ | absent | BT9727_2890 | absent | BC3101* | absent |  |
| *hlyII* | hemolysin II | frameshift | BT9727_2311 | BCE33L2269 | BC3523 | BACI_c24970 | N-terminal part absent in Ba |
| *hlY-III* (*yplQ*) | hemolysin III | GBAA_2241 | BT9727_2027 | BCE33L2025 | BC2196 | BACI_c21980 |  |
| *nheA* | non-hemolytic enterotoxin lytic component L2 | GBAA_1887 | BT9727_1727 | BCE33L1698 | BC1809* | BACI_c18740 |  |
| *nheB* | non-hemolytic enterotoxin lytic component L1 | GBAA_1888 | BT9727_1728 | BCE33L1699 | BC1810* | BACI_c18750 |  |
| *nheC* | enterotoxin C | premature stop codon | BT9727_1729 | BCE33L1700 | BC1811* | BACI_c18760 | C-terminal part absent in *B. anthracis* Ames Ancestor |
| *clo* (*alo*) | thiol-activated cytolysin (cereolysin O, anthrolysin O) | GBAA_3355 | BT9727_3096 | BCE33L2999 | BC5101* | BACI_c32520 |  |
| *calY1* | camelysin, cell envelope-bound metalloprotease | GBAA_1290 | BT9727_1173 | BCE33L1175 | BC1281 | BACI_c13150 |  |
|  | camelysin | absent | absent | absent | absent | BACI_c06480 |  |
|  | camelysin | absent | absent | BCE33L4622 | BC4868 | BACI_c48740 |  |
| *plcA* | phosphatidylinositol-specific phospholipase C | GBAA_3891 | BT9727_3501 | BCE33L3513 | BC3761* | BACI_c37040 |  |
| *plcB* (*cerA*) | phosphatidylcholine-specific phospholipase C | GBAA_0677 | BT9727_0587 | BCE33L0588 | BC0670* | BACI_c06850 |  |
| *sph* (*cerB*) | sphingomyelinase | GBAA_0678 | BT9727_0588 | BCE33L0589 | BC0671* | BACI_c06860 |  |
| *cytK* | cytotoxin K | absent | BT9727_1008 | BCE33L1009 | BC1110* | absent |  |
| *colA* | microbial collagenase | GBAA_3299 | absent | pE33L466_0145 | BC3161* | absent | *B. cereus* E33L: plasmid-encoded |
| *colC* | microbial collagenase | GBAA_0555 | BT9727_0466 | BCE33L0466 | BC0556 | BACI_c05630 |  |
| *sfp* | serine protease | GBAA_3892 | BT9727_3502 | BCE33L3514 | BC3762* | BACI_c37050 |  |
| *colA* | microbial collagenase | GBAA_3584 | BT9727_3286 | BCE33L3238 | BC3529 | BACI_c34720 |  |
| *mpbE* | enhancin protein, metalloprotease | GBAA_3443 | BT9727_3171 | BCE33L3092 | BC3384* | BACI_c33440 |  |
| *npr2* | neutral protease (bacillolysin) | GBAA_3442 | BT9727_3170 | BCE33L3091 | BC3383* | BACI_c33430 |  |
| *nprE2* | Bacillolysin, neutral subtilase | GBAA_2730 | BT9727_2499 | BCE33L2464 | BC2735* | BACI_c26950 |  |
| *pnrE1* | bacillolysin | absent | BT9727_2348 | BCE33L2310 | BC2506 | BACI_c25380 |  |
| *nprB* | neutral protease B (bacillolysin) | absent | absent | absent | BC5351* | BACI_c50530 |  |
| *inhA1* | immune inhibitor A1, metalloprotease | GBAA_1295 | BT9727_1175 | BCE33L1177 | BC1284 | BACI_c13170 |  |
| *inhA2* | immune inhibitor A2, metalloprotease | GBAA_0672 | BT9727_0582 | BCE33L0581 | BC0666* | BACI_c06810 |  |
| *inlA* | internalin | GBAA_1346 | BT9727_1220 | BCE33L1222 | BC1331 | BACI_c13660 | N-terminus truncated in Ba and CI |
|  | internalin | GBAA_0552 | BT9727_0463 | BCE33L0459 | BC0552 | BACI_c05600 | weak homologies, see Table S4 |
| *bla1* | beta-lactamase | GBAA_2507 | BT9727_2291 | BCE33L2247 | BC2473 | BACI_c24760 |  |
| *bla2* | beta-lactamase II | GBAA_3500 | BT9727_3218 | BCE33L3155 | BC3440 | BACI_c33980 | N-terminus truncated in *B. cereus* E33L |
| *lmrB* | lincomycin resistance protein | absent | absent | BCE33L0734 | absent | BACI_c08770 |  |
|  | lincomycin resistance protein | GBAA_0944 | absent | absent | BC0962 | BACI_c09790 |  |
|  | drug resistance transporter | GBAA_1038 | BT9727_0959 | BCE33L0949 | absent | BACI_c10760 |  |
| *mrsR* | response regulator | absent | BT9727_0923 | BCE33L0906 | absent | BACI_c10360 | mersacidin resistance operon |
| *mrsK* | sensor histidine kinase | absent | BT9727_0924 | BCE33L0907 | absent | BACI_c10370 |  |
| *mrsF* | ABC transporter, ATP binding protein | absent | BT9727_0925 | BCE33L0908 | absent | BACI_c10380 |  |
| *mrsG* | ABC transporter, permease | absent | BT9727_0926 | BCE33L0909 | absent | BACI_c10390 |  |
| *mrsE* | permease | absent | BT9727_0927 | BCE33L0910 | absent | BACI_c10400 |  |
| *salX* | ABC transporter, ATP binding protein | absent | BT9727_2956 | BCE33L2892 | absent | BACI_c31330 | salivaricin resistance operon |
| *salY* | ABC transporter, permease | absent | BT9727_2957 | BCE33L2893 | absent | BACI_c31340 | N-terminus shortened |
| *salK* | sensor histidine kinase | absent | BT9727_2958 | BCE33L2894 | absent | BACI_c31350 |  |
| *salR* | response regulator | absent | BT9727_2959 | BCE33L2895 | absent | BACI_c31360 |  |
| *plcR* | transcriptional regulator PlcR | premature stop codon | BT9727_5033 | BCE33L5049 | BC5350* | BACI_c53450 | C-terminus altered in CI |
| *papR* | PapR protein | GBAA_5594 | BT9727_5032 | BCE33L5048 | BC5349* | BACI_c53440 |  |
|  | transcriptional regulator (PlcR-paralog) | GBAA_1163 | BT9727_1061 | BCE33L1058 | BC1158 | BACI_c11960 |  |
|  | PlcR-paralog | absent | absent | pE33L466_0425 | BC0988 | absent | *B. cereus* E33L: plasmid-encoded |
|  | PlcR-paralog | absent | absent | absent | BC2443 | absent |  |
| *nprR* | transcriptional regulator (PlcR family protein) | GBAA_0597 | BT9727_0508 | BCE33L0510 | BC0598 BC0601 | BACI_c06070 | gene interrupted by IS element in *B. cereus* 14579 |
| *abrB* | transition state regulator | GBAA_0034 | BT9727_0033 | BCE33L0033 | BC0042 | BACI_c00440 |  |
| *abrB* | transition state regulator | GBAA_2000 | BT9727_1828 | BCE33L1812 | BC1996 | BACI_c19730 |  |
| *pagA* | protective antigen | GBAA_pXO1_0164 | absent | absent | absent | BACI_pCIXO101620 |  |
| *lef* | lethal factor | GBAA_pXO1_0172 | absent | absent | absent | BACI_pCIXO101690 |  |
| *cya* | edema factor (calmodulin-sensitive adenylate cyclase) | GBAA_pXO1_0142 | absent | absent | absent | BACI_pCIXO101370 |  |
| *bslA* | *B. anthracis* S-layer protein A (BslA) adhesin | GBAA_pXO1_0124 | absent | absent | absent | BACI_pCIXO101160 | C-terminus altered (5 aa) and extended (21 aa) in CI due to frameshift |
| *atxA* | transcriptional activator AtxA | GBAA_pXO1_0146 | absent | absent | absent | BACI_pCIXO101440 |  |
| *pagR* | transcriptional repressor PagR | GBAA_pXO1_0166 | absent | absent | absent | BACI_pCIXO101640 |  |
| *capB* | capsule biosynthesis protein CapB (mur ligase) | GBAA_pXO2_0066 | absent | absent | absent | BACI_pCIXO200640 |  |
| *capC* | capsule biosynthesis protein CapC | GBAA_pXO2_0065 | absent | absent | absent | BACI_pCIXO200630 |  |
| *capA* | capsule biosynthesis protein CapA | GBAA_pXO2_0064 | absent | absent | absent | BACI_pCIXO200620 |  |
| *capD* (*dep*) | capsule biosynthesis protein CapD (gamma-glutamyltranspeptidase) | GBAA_pXO2_0063 | absent | absent | absent | BACI_pCIXO200610 |  |
| *capE* | capsule biosynthesis protein CapE | GBAA_pXO2_0062 | absent | absent | absent | BACI_pCIXO200600 |  |
| *acpA* | capsule biosynthesis regulator AcpA | GBAA_pXO2_0084 | absent | absent | absent | BACI_pCIXO200830 |  |
| *acpB* | capsule biosynthesis regulator AcpB | GBAA_pXO2_0060 | absent | absent | absent | BACI_pCIXO200580 |  |

* PlcR-regulation confirmed in *B. cereus* ATCC 14579 (Gohar et al., ref. 56)
