# Supplementary material for: The Genome of a Bacillus Isolate Causing Anthrax in Chimpanzees Combines Chromosomal Properties of B. cereus with B. anthracis Virulence Plasmids
Source: PLoS One. 2010 Jul 9;5(7):e10986. doi: 10.1371/journal.pone.0010986 (PMC2901330; doi:10.1371/journal.pone.0010986)
Supplement: Table S4 — Identity of internalin proteins present at comparable genome positions. (0.03 MB DOC) [file pone.0010986.s008.doc]

**Table S4.** Identity of internalin proteins present at comparable genome positions.

|  | BA0552 | BT9727_0463 | BCZK0459 | BC0552 | BACI_c05600 |
| --- | --- | --- | --- | --- | --- |
| BA0552 | - | 44 % | 92 % | 52 % | 54 % |
| BT9727_0463 |  | - | 50 % | 67 % | 52 % |
| BCZK0459 |  |  | - | 53 % | 52 % |
| BC0552 |  |  |  | - | 56 % |
| BACI_c05600 |  |  |  |  | - |
